# Neutrophil gelatinase-associated lipocalin in ICU patients developing oliguria

**DOI:** 10.1186/cc9527

**Published:** 2011-03-11

**Authors:** A Roman, M Suball, V Piersoel, T El Mahi, C Hanicq, E Stevens

**Affiliations:** 1 CHU Saint-Pierre, Brussels, Belgium

## Introduction

Plasma neutrophil gelatinase-associated lipocalin (pNGAL) is an early biomarker of acute kidney injury (AKI) [1].

## Methods

A prospective observational study enrolling adult ICU patients developing a first episode of oliguria defined as urinary output lower than 0.5 ml/kg/hour for at least only 2 consecutive hours despite conventional treatment and appropriate fluid resuscitation. pNGAL (Biosite, Inverness, San Diego, CA, USA), plasma cystatin C, plasma and urinary sodium and creatinine, were measured to determine on 1 hour the fraction of excretion of the filtered sodium (FeNa) and the glomerular filtration rate (GFR). The SOFA score and RIFLE score [2] were calculated. Hospital mortality was recorded.

## Results

Ninety-three patients were enrolled: 52 presented with 0, 15 with R, 13 with I and 10 with F RIFLE score. The median SOFA score was 3 (minimum: 0 to maximum: 17). Sepsis was the main diagnostic in 38 patients, 27 were cardiac surgery patients who underwent cardiopulmonary bypass (CBP) and 28 were miscellaneous other category patients (hemorrhagic shock, hypotensive surgery, trauma with crush, and so on). In-hospital mortality of the studied cohort was 20%. Eighty-five percent of FeNa were less than 1%, suggesting active antidiuresis and sodium reabsorption. The distribution of pNGAL between survivors (median 61 ng/ml, 95% CI = 59 to 91 ng/ml) and nonsurvivors (median 182 ng/ml, 95% CI = 86 to 594 ng/ml) was statistically significant (*P *= 0.006, Wilcoxon rank test). Distribution of pNGAL in patients post CPB (median 59 ng/ml; 95% CI = 59 to 59), was statistically different from patients with sepsis (median 180 ng/ml; 95% CI = 92 to 276) and the last group (median 85 ng/ml; 95% CI = 59 to 166) with respectively *P *< 0.0001 and 0.024 after Bonferroni's correction. No correlation between pNGAL and FeNa was found (Spearman's rho = 0.309; 95% CI = 0.11 to 0.48), nor between pNGAL and 1-hour GFR (Spearman's rho = -0.55; 95% CI = -0.68 to -0.38), neither between pNGAL and plasma cystatin C (Spearman's rho = 0.62; 95% CI = 0.47 to 0.73).

## Conclusions

pNGAL rises in early oliguria independently while kidney function markers such as GFR, FeNa and cystatin C may have remained unaffected at this stage. Sepsis is a stronger trigger for pNGAL elevation.
